# Marine heatwaves suppress nutrient utilization and reproductive flexibility in humpback whales

**DOI:** 10.1016/j.isci.2026.117181

**Published:** 2026-08-19

**Authors:** Ching-Tsun Chang, Cody W. Clifton, Kristi L. West, Debbie Steel, Scott Baker, Natalie J. Wallsgrove, Brian N. Popp

**Affiliations:** 1 Department of Oceanography, University of Hawaiʻi at Mānoa, Honolulu, HI 96822, USA; 2 Eastern Fishery Research Center, Fisheries Research Institute, Taitung 961, Taiwan; 3 Department of Oceanography, National Sun Yat-sen University, Kaohsiung, 80424, Taiwan; 4 Health and Stranding Lab, Human Nutrition, Food and Animal Sciences, College of Tropical Agriculture and Human Resilience, University of Hawaiʻi at Mānoa, Honolulu, HI 96822, USA; 5 Marine Mammal Institute, Oregon State University, Corvallis, OR 97365, USA; 6 Department of Earth Sciences, University of Hawaiʻi at Mānoa, Honolulu, HI 96822, USA

**Keywords:** Metabolic flexibility, Compound-specific isotope analysis, Capital breeding, Reproductive energetics, Protein turnover, Megaptera novaeangliae, Bioenergetic modeling

## Abstract

Marine heatwaves (MHWs) are intensifying with climate change, raising questions about how extreme thermal events alter the physiology of large migratory mammals. We reconstructed eight years of continuous metabolic histories from baleen of three female humpback whales using compound-specific amino acid isotope analyses integrated with bioenergetic models. These records reveal pronounced shifts in nutrient allocation and metabolic indicators during the 2014–2016 MHW, with model outputs predicting reduced field metabolic rates marked by suppressed protein turnover and diminished lipid use. In contrast, non-MHW periods showed more efficient nutrient routing that supported elevated metabolic demands. Pregnant females were predicted to exhibit constrained metabolic flexibility during MHWs, and isotopic patterns following the event suggest energetic limitations consistent with unsuccessful early pregnancies or pseudopregnancies. These results illustrate how climate-driven extremes and reproductive demands interact to shape metabolic strategies in humpback whales, underscoring vulnerabilities in capital breeding species as MHWs become more frequent.

## Highlights

• MHWs trigger metabolic downregulation in humpback whales to conserve protein
• Capital breeders show reduced metabolic flexibility during MHWs
• Isotopic evidence links energetic constraints to unsuccessful early pregnancies
• CSIA-AA and DNA reveal decadal physiological shifts in a 27-year-old whale

## Graphical abstract

**Figure:**
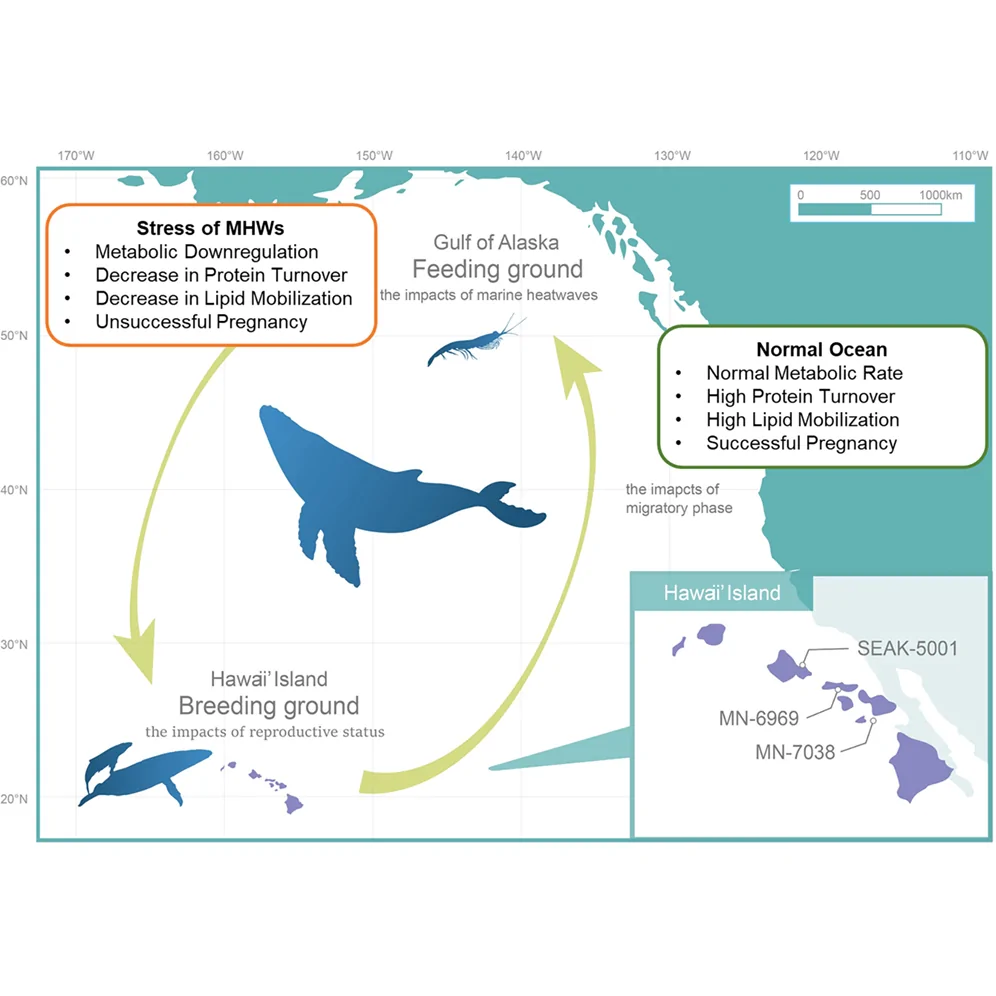

## Teaser

Marine organism; Environmental science; Ecology; Zoology

## Introduction

Marine heatwaves (MHWs), extreme warming events, are intensifying with climate change, and driving abrupt shifts in prey availability, food-web structure, and ecosystem productivity.^1^^,^^2^^,^^3^ MHWs are defined as discrete periods of anomalously warm sea temperatures exceeding a seasonally varying 90^th^-percentile threshold, with intensity classified from moderate to extreme depending on the magnitude of the anomaly.^4^^,^^5^ These events pose substantial bioenergetic challenges for marine endotherms by simultaneously elevating metabolic demands and reducing energy intake through declines in lower trophic level productivity.^6^^,^^7^ For example, elevated temperatures have been associated with reduced foraging success and lower reproductive effort in blue whales during an MHW.^8^ For large migratory capital breeders such as humpback whales (*Megaptera novaeangliae*), which rely on energy acquired and stored during seasonal foraging to sustain migration, pregnancy, and lactation,^9^^,^^10^^,^^11^ MHWs may impose significant physiological pressure, particularly on pregnant and lactating females. Although humpback whales exhibit flexible foraging strategies, they often reduce or cease feeding during specific migratory or reproductive phases,^12^ increasing their vulnerability to climate-driven disruptions.

One of the most extreme events, the 2014–2016 “Blob” MHW, produced anomalously warm sea surface temperatures across the Pacific Ocean, with especially severe impacts in the northeast.^13^ This event coincided with pronounced declines in the abundance, biomass, and distribution of zooplankton, including krill and forage fishes,^14^ and was followed by reduced sightings of adult humpback whales and calves.^15^ When prey availability declines, whales may reduce metabolic rates to lower total energy demand. However, even with such downregulation, individuals must rely heavily on endogenous energy reserves to sustain maintenance and reproduction, an energy trade-off documented in other marine mammals.^16^ In most species, physiological maintenance is prioritized over reproductive investment when energy becomes limited, often resulting in reduced, delayed, or suspended reproductive effort.^7^^,^^8^ Yet, the mechanisms by which episodic environmental disturbances influence nutrient utilization and metabolic allocation in whales remain poorly understood.

Reproductive status imposes additional metabolic demands and can shape an individual’s capacity to cope with environmental stress. Gestation and subsequent lactation draw heavily on endogenous lipid and protein stores,^7^^,^^17^ influencing the metabolic buffer available to cope with environmental stressors during MHWs. Progesterone preserved in baleen provides a robust, long-term record of reproductive transitions, enabling pregnancies, and associated physiological shifts to be identified with high confidence, particularly when interpreted alongside androstenedione, 17β-estradiol, and testosterone.^18^^,^^19^^,^^20^^,^^21^^,^^22^^,^^23^^,^^24^^,^^25^ Integrating hormone-based reproductive histories with biochemical tracers allows reconstruction of the interactive effects of reproductive demands and environmental variability on the metabolic strategies of free-ranging whales.

In marine mammals, prolonged fasting (fasting stage II) typically involves a transition from glucose to lipid-derived fuels while minimizing protein catabolism to maintain homeostasis.^26^^,^^27^ Under extreme nutritional stress or lipid depletion (stage III), individuals rely on endogenous protein to sustain the essential metabolic functions.^28^^,^^29^ Compound-specific isotope analysis of amino acids (CSIA-AA) provides a powerful approach to disentangle dietary from metabolic processes in shaping nitrogen balance.^30^ Trophic amino acids (e.g., glutamic acid, alanine, and proline), serve as metabolic indices because they exchange nitrogen through transamination and become enriched in ^15^N during metabolic activity. In contrast, source amino acids (e.g., phenylalanine and lysine) retain environmental baseline δ^15^N values, with minimal ^15^N enrichment from metabolism. The δ^15^N offsets between these amino acid groups provide estimates of trophic position and illuminate metabolic strategies, revealing the relative contributions of exogenous dietary intake versus endogenous protein catabolism.^31^ During fasting, reliance on gluconeogenesis elevates δ^15^N values in gluconeogenic amino acids (glycine, serine, proline, and aspartic acid) due to deamination and recycling through the metabolic nitrogen pool.^31^^,^^32^ Carbon isotope analyses of amino acids offer complementary insights into metabolic routing. Essential amino acids, such as phenylalanine, largely retain dietary δ^13^C values, whereas non-essential amino acids (e.g., glutamic acid, aspartic acid, and alanine) can be synthesized *de novo* and thus incorporate carbon derived from lipid, carbohydrate, or protein.^17^ Under conditions of lipid mobilization, non-essential amino acids tend to become more ^13^C-depleted.^17^ Thus, compound-specific isotope records of nitrogen and carbon archived in baleen plates provide a window into the interplay between environmental stress, reproductive status, and metabolic allocation in free-ranging whales.

Baleen is composed of keratin and grows slowly, providing a stable tissue that preserves a chronological record of an individual’s physiology and behavior.^33^^,^^34^ As it grows, baleen incorporates biochemical signals that reflect natural behaviors and developmental changes.^33^^,^^34^^,^^35^ Serial sampling along the growth axis has been widely used to examine isotopic and hormonal variation in adult whales.^34^^,^^36^ These studies have shown that baleen biochemistry remains stable for decades at room temperature, enabling retrospective analyses from archival specimens.

**Figure 1:**
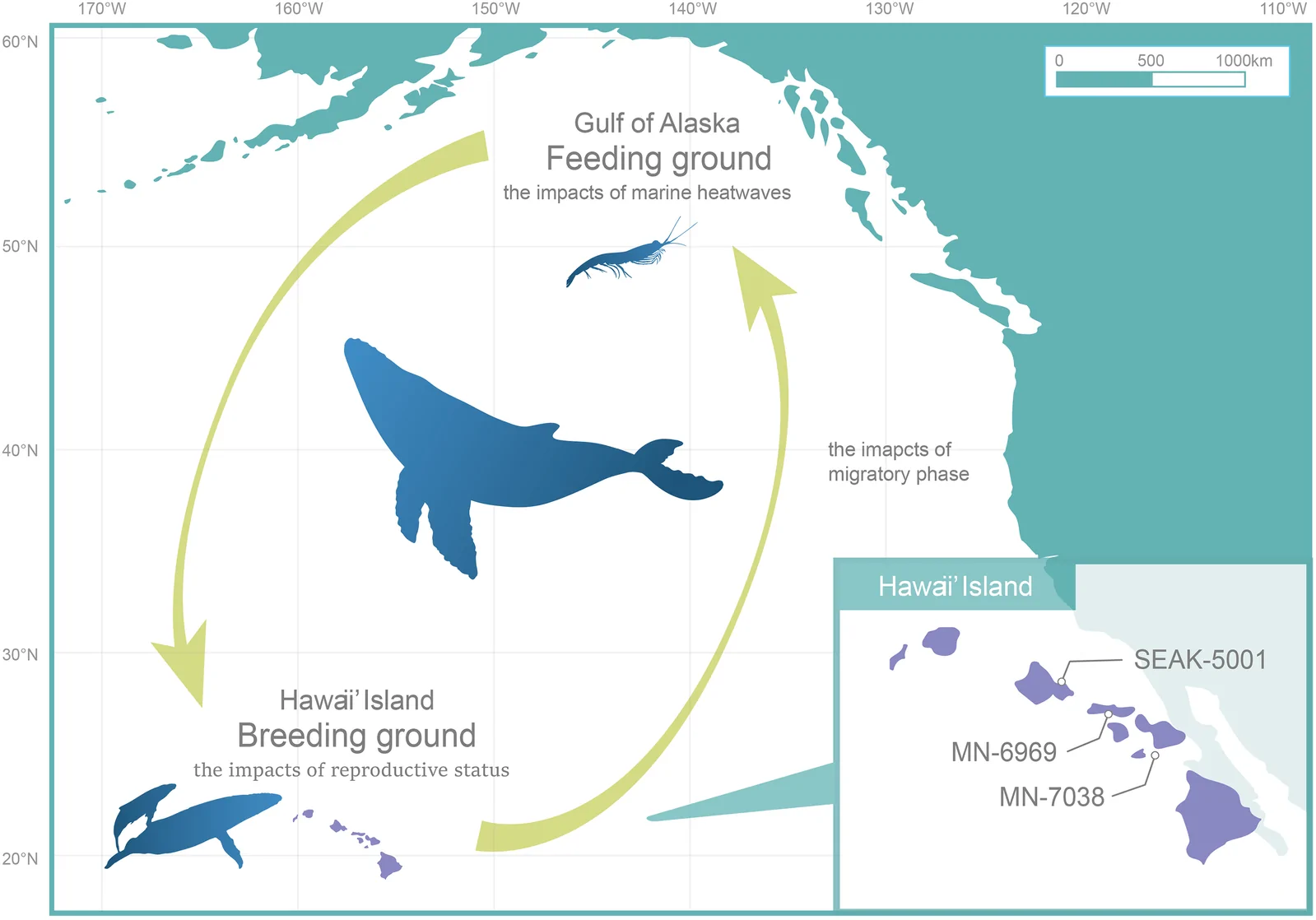
Schematic of the study system and hypotheses Schematic showing the migratory pathway of humpback whales (*Megaptera novaeangliae*) between Hawaii (breeding grounds) and the Gulf of Alaska (feeding grounds), illustrating the hypothesized influences of reproductive status, migratory phase, and marine heatwaves (MHWs) on whale metabolism and nutrient allocation. Green arrows indicate the typical migration route between foraging and breeding regions. Sampling locations of the three stranded female humpback whales analyzed in this study (MN-7038, MN-6969, and SEAK-5001) are shown around the Hawaiian Islands.

In this study, we coupled baleen isotopic records with progesterone profiles from three stranded female humpback whales to investigate how exposure to MHWs, reproductive status, and migratory phase influence nutrient utilization strategies (Figure 1). We hypothesized that (1) MHWs reduce trophic position by lowering δ^15^N values in source amino acids and diminishing trophic ^15^N enrichment in glutamic acid, consistent with shortened food-chain length during heatwave conditions; (2) migratory phase influences nutrient utilization, with whales on feeding grounds exhibiting higher δ^15^N values of bulk baleen and of glucogenic amino acids and lower δ^13^C values in non-essential amino acids in breeding grounds due to increased reliance on lipid stores; (3) pregnancy alters metabolic routing by promoting protein turnover and lipid reliance, resulting in increased ^15^N enrichment in glucogenic amino acids and greater ^13^C-depletion in non-essential amino acids compared with non-pregnant whales; and (4) reproductive status modulates the metabolic response to environmental stress, with non-pregnant individuals exhibiting greater metabolic flexibility and stronger isotopic evidence of altered nutrient routing and endogenous reserve use. By linking ecosystem perturbations to individual physiological responses, this study provides insight into how climate extremes may influence the bioenergetics of migratory megafauna.

## Results

### Genetic profiling of stranded humpback whales

The DNA profile generated from the whale that stranded at Waimanalo, Oʻahu, on April 12, 2021, matched a previously sampled individual within the DNA register, identifying her as Sprinter/Helleborine (SEAK-5001; Figure 1). This whale was first sampled and photographed during the 2004–2006 SPLASH study, which paired fluke photographs with genetic profiles from biopsies collected across the North Pacific.^8^^,^^37^ Historic fluke photographs archived in the Happywhale database further identified SEAK-5001 on August 19, 1993, in waters off Southeast Alaska and British Columbia^38^ (photo credit: Fred Sharpe), indicating she was more than 28 years old at the time of death.

Happywhale records document 15 total sightings between 1993 and 2021, placing SEAK-5001 in coastal Alaska, British Columbia, and Hawaii, including multiple encounters in the month preceding the discovery of her carcass off Oʻahu and subsequent stranding at Waimanalo. On March 25, 2021, SEAK-5001 was photographed near Lahaina, Maui, within a competitive group of approximately six whales, with images showing a large lesion on the top and right side of the head, posterior to the blowholes (photo credit: James Begeman). Additional photographs from March 30 and April 1, 2021, documented the wound and high levels of surface activity, including pectoral fin slapping (photo credit: James Begeman). Final fluke photos were obtained off Maui on April 2, ten days prior to her stranding on Oʻahu (photo credits: Anusha Mathur, Pacific Whale Foundation; S. Bryner, Hawaiian Islands Humpback Whale National Marine Sanctuary). Genetic profiles generated from the other two stranded individuals did not match any previously sampled whales in the DNA register.

### Bulk isotope records reveal long-term variation across baleen

**Figure 2:**
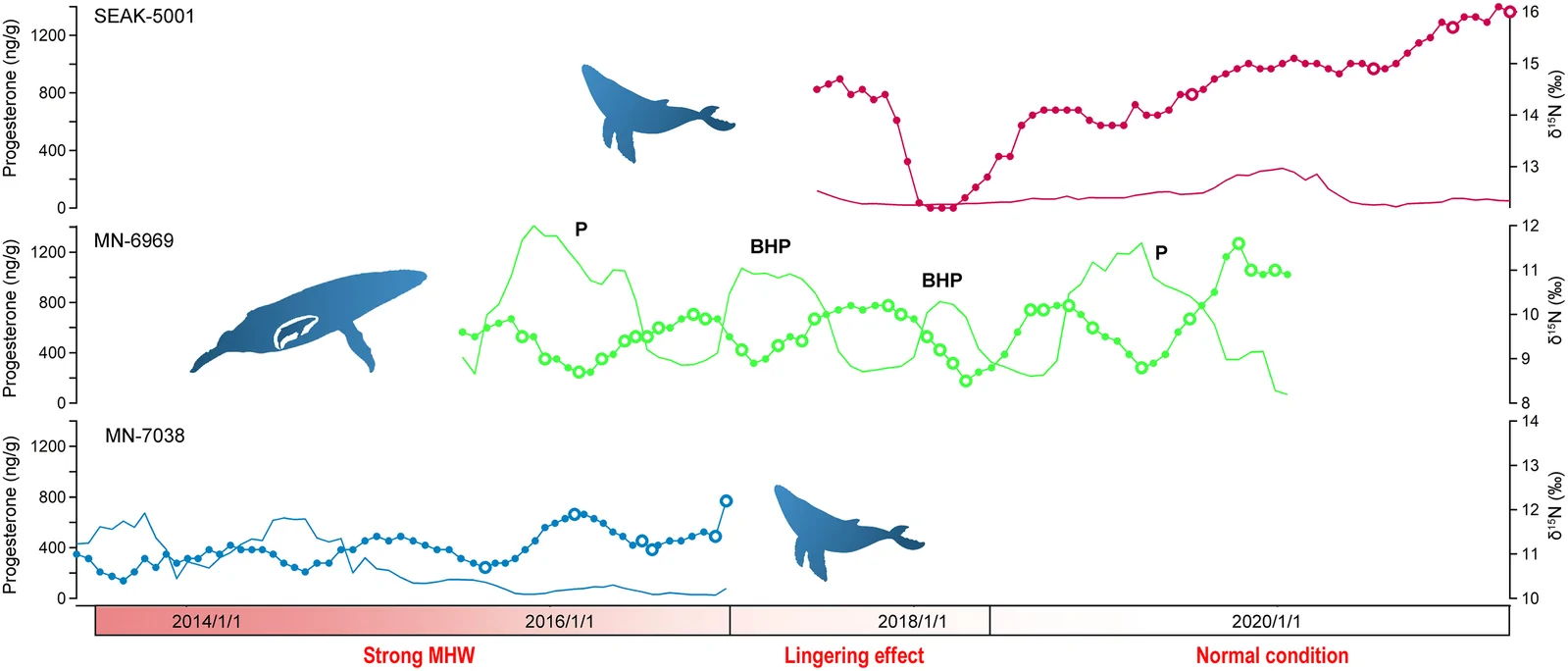
Temporal variation in progesterone concentrations and bulk δ^15^N values in baleen from three female humpback whales Time-series plots show progesterone concentrations (solid lines) and bulk δ^15^N values (line with circles) measured along the baleen plates of three stranded females (blue, MN-7038; green, MN-6969; and red, SEAK-5001). White open circles on the δ^15^N profiles denote samples selected for compound-specific isotope analysis of amino acids. Inferred reproductive stages are annotated for MN-6969, including two full-term pregnancies (P) and two brief high-progesterone (BHP) periods. SEAK-5001 and MN-7038 were categorized as non-pregnant throughout their records. The red-to-white gradient at the base of the plot represents the impacts of the 2014–2016 marine heatwave (MHW), transitioning from strong environmental stress to lingering effects and subsequent normal oceanographic conditions.

Basic information for the three-stranded humpback whales is provided in Table 1. Bulk δ^15^N values ranged from 8.5‰ to 16.1‰ across individuals (Figure 2). Two whales (MN-6969 and MN-7038) exhibited clear oscillatory patterns consistent with repeated movement between feeding and breeding ground, with significantly higher δ^15^N values for each individual during presumed breeding ground periods and lower values during feeding ground periods (Wilcoxon test: *p* < 0.001, *n* = 60 for MN-6969 and *n* = 72 for MN-7038). In contrast, bulk zooplankton δ^15^N values showed the opposite pattern. Mixed zooplankton collected from the upper 150 m at Station ALOHA, 100 km north of Oʻahu,^39^ had significantly lower δ^15^N values (*p* < 0.001, *n* = 71) than zooplankton collected from similar depths at Station Papa, ∼1,750 km west of Vancouver Island in the Gulf of Alaska.^40^ The baleen records collectively captured nearly eight years of isotopic chronology (5/2013–4/2021). Bulk isotope data for MN-6969 were reproduced from Clifton et al. (unpublished data) for comparison (Figure 2).

**Table 1:** The summary information of three stranded female humpback whales

Animal ID	Date of stranding	Stranding location	Total length (cm)	Approximate baleen length
MN-7038	2016/12/29	ʻĀhihi, Maui	Unknown	∼65 cm
MN-6969	2020/1/21	Hale O Lono, Molokaʻi	1341	∼75 cm
SEAK-5001	2021/4/12	Waimanalo, Oʻahu	1030	∼65 cm

### Reproductive status determined from progesterone and amino acid δ^15^N

Progesterone concentrations from MN-6969, MN-7038, and SEAK-5001 revealed distinct periods corresponding to non-pregnant states, brief progesterone elevation, and sustained high progesterone levels consistent with pregnancy (Figure 2). As detailed in the STAR Methods, pregnant status was assigned to intervals with elevated progesterone maintained for >11 months; non-pregnant states were identified by concentrations near baseline; and brief-duration progesterone elevations were characterized by short-lived increases above baseline.

The three individuals differed in the degree of reproductive confirmation. MN-6969 provided direct validation of the progesterone-inferred pregnancy through the recovery of a near full-term fetus at the time of death, which aligned with a prolonged high progesterone interval. A presumed earlier pregnancy was also identified in MN-6969 based on similar hormonal and isotopic signatures. Two brief progesterone elevations in MN-6969 between 2017 and 2018, lasting approximately 9 and 6 months, respectively, are durations shorter than expected for full-term gestation. In contrast, reproductive status for MN-7038 was inferred solely from progesterone profiles, as no secondary confirmation (e.g., fetal recovery) was available. SEAK-5001 exhibited only baseline progesterone concentrations throughout the baleen record, consistent with non-pregnant status.

Across all individuals, δ^15^N values of AAs differed between pregnant and non-pregnant intervals. Subsequent principal-component analysis (PCA) and linear discriminant analysis (LDA) distinguished brief progesterone-elevation periods as a second cluster, clearly differentiated from both confirmed pregnant and non-pregnant stages (Figure S1).

### Dominant factors shaping baleen isotope variation

**Figure 3:**
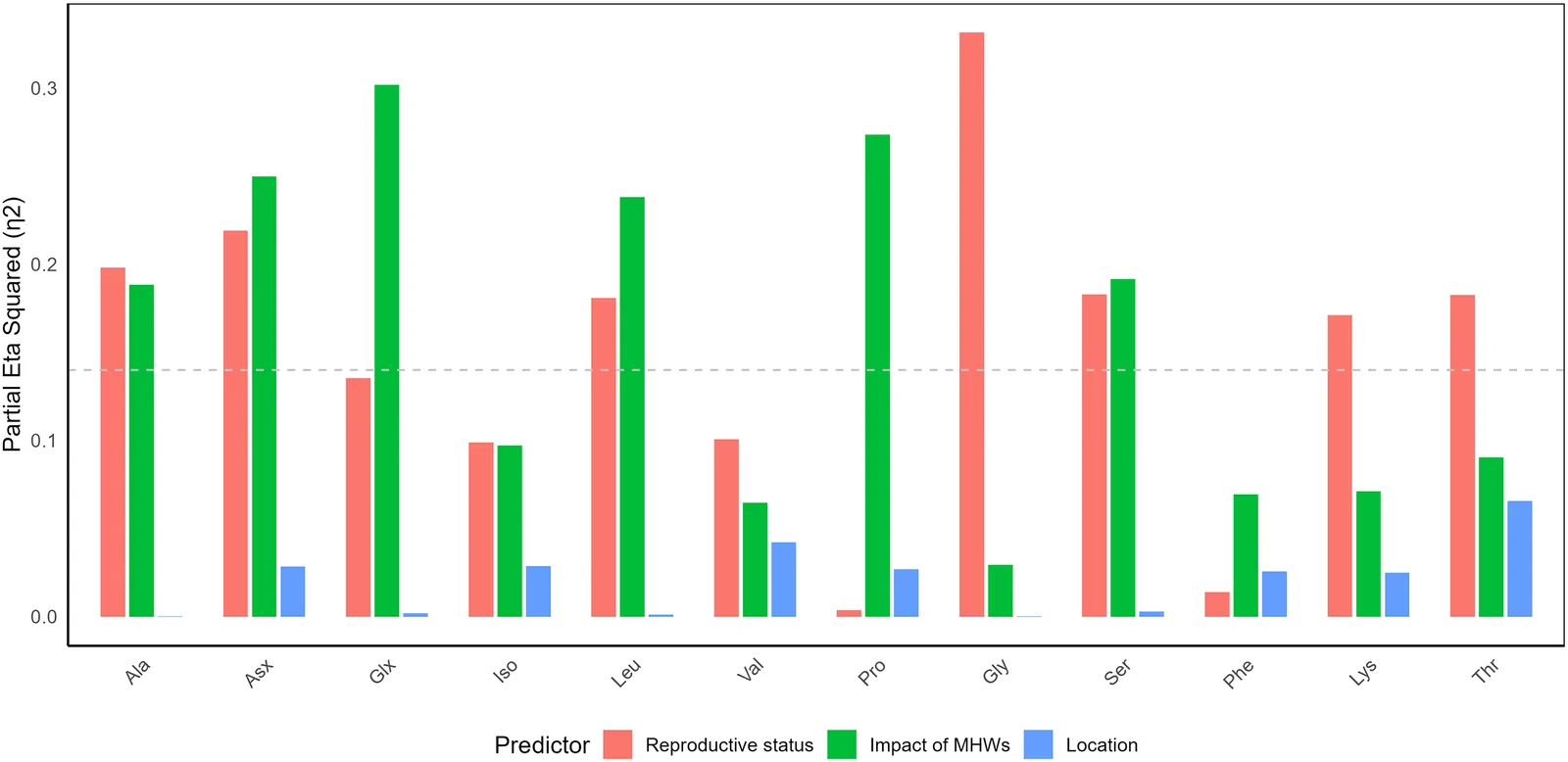
Partial η^2^ effect sizes for factors influencing δ^15^N values of AAs in humpback whale Bars show the variance explained by MHWs (green), reproductive status (red), and location (blue) for each AA; the dashed line marks η^2^_partial = 0.14, the conventional threshold for a large effect. Across AAs, MHWs and reproductive status exhibited larger effect sizes than location.

We quantified the relative influence of environmental stress, reproductive status, and migratory phase on baleen δ^15^N values using partial η^2^ effect sizes. Environmental stress was categorized by MHW intensity (normal years, strong MHWs, and lingering post-MHW periods). Both MHW exposure and reproductive status explained substantial proportions of variance across amino acids, whereas location effects were negligible (Figure 3).

MHWs exerted the strongest influence on most trophic amino acids (i.e., aspartic acid, leucine, and proline), while reproductive status accounted for greater variation in glycine, lysine, and threonine. Consistent with these patterns, multivariate analyses of amino acid δ^15^N values showed substantial overlap between feeding- and breeding-ground samples, regardless of whether analyses included only source amino acids or all amino acids (Figure S2), even though bulk baleen δ^15^N values were different between feeding- and breeding-ground samples.

### MHWs reshape nutrient routing and metabolism

**Figure 4:**
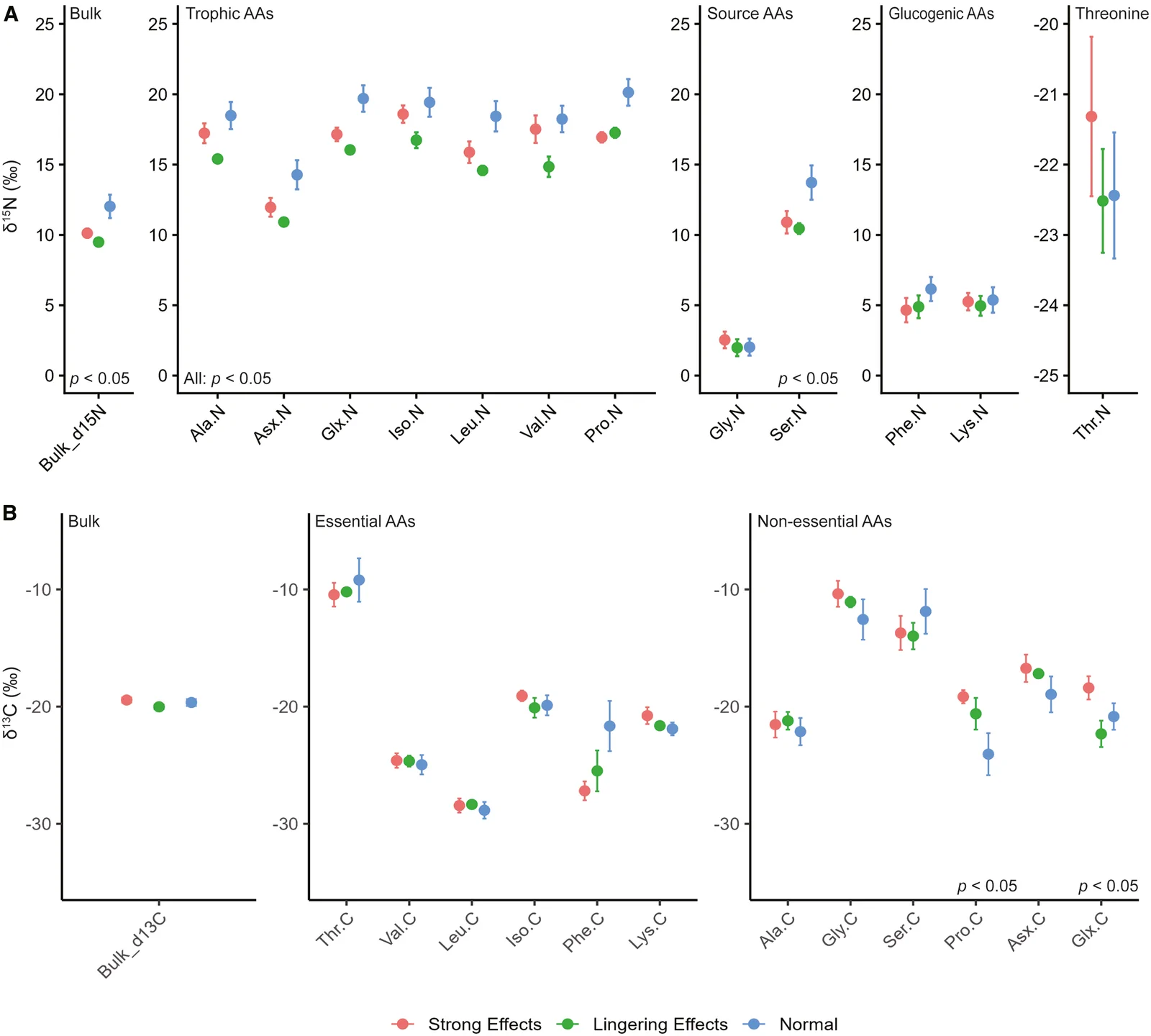
Variations in δ^15^N and δ^13^C values of bulk tissue and amino acids under environmental stress (A and B) During normal periods, δ^15^N values of trophic amino acids, bulk tissue, and serine (glucogenic amino acid) were consistently higher than those measured during marine heatwaves and lingering effects (A). In contrast, most δ^13^C values of bulk tissue and amino acids did not differ significantly among the environmental stress (B). Data are represented as mean δ^15^N or δ^13^C values ± SE.

Bulk and trophic amino acid δ^15^N values declined significantly during strong and lingering MHW periods relative to normal years (Figure 4A and Table S1). In contrast, δ^13^C values of bulk baleen and most amino acids did not differ across MHW categories (Figure 4B and Table S2).

To evaluate shifts in nutrient routing and metabolic strategy, we calculated several isotopic indices (see STAR Methods). Metabolic indices revealed clear differences between normal and heatwave conditions. The difference between δ^15^N values of glutamic acid and phenylalanine (Δ^15^N_Glx–Phe_) reflects effective food-chain length and trophic position.^41^ The gluconeogenic (GNG) index represents the offset between mean δ^15^N values of primary gluconeogenic substrates and baseline. The trophic metabolic index (TMI) captures the offset between δ^15^N values of trophic amino acids strongly involved in the metabolic nitrogen pool and source amino acids with minimal metabolic fractionation. The carbon metabolic index (CMI) reflects the δ^13^C difference between non-essential amino acids and essential amino acids. CMI was calculated for two individuals because δ^13^C values were unavailable for SEAK-5001 during normal years.

**Figure 5:**
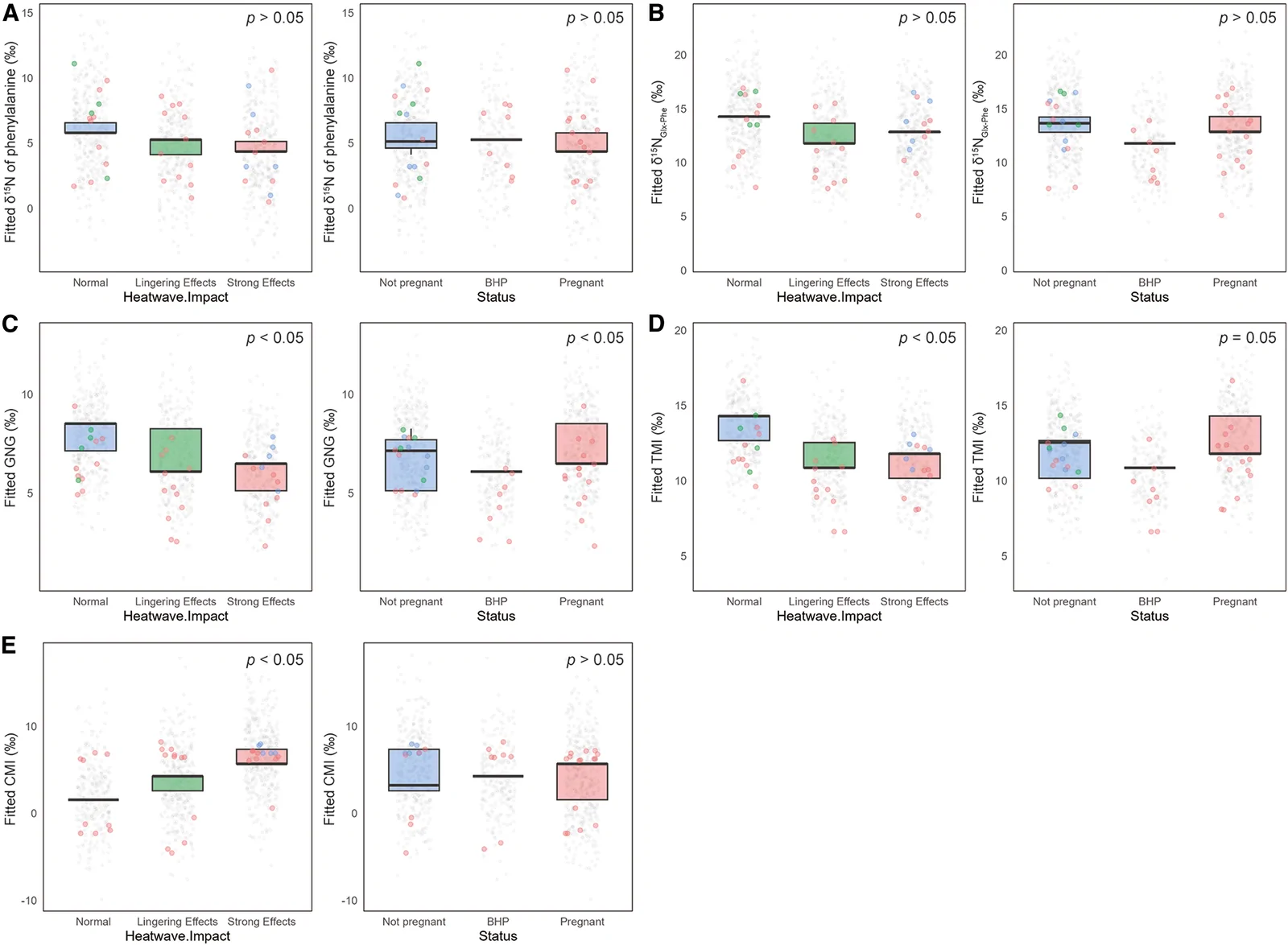
Variations in metabolic and isotopic indices across reproductive states and marine heatwave conditions (A–E) Linear mixed model results showing differences in (A) δ^15^N values of phenylalanine, (B) Δ^15^N_Glx–Phe_, (C) the gluconeogenic index (GNG), (D) trophic metabolic index (TMI), and (E) carbon metabolic index (CMI) across reproductive states and marine heatwave categories. Colored points represent observations from individual whales (green, SEAK-5001; red, MN-6969; and blue, MN-7038), whereas gray points indicate simulated values generated by bootstrap resampling from the fitted models. Boxplots represent fitted values from fixed effects of the linear mixed models. *p* values indicate significance of fixed effects.

Phenylalanine δ^15^N values showed a slight but non-significant decline during strong and lingering MHW, consistent with a modest baseline lightening effect (*χ*^*2*^ = 2.464, *p* = 0.292, *n* = 38; Figure 5A). Similarly, Δ^15^N_Glx–Phe_ did not differ significantly across MHW categories (*χ*^*2*^ = 1.743, *p* = 0.418, *n* = 38; Figure 5B). In contrast, both GNG (*χ*^*2*^ = 20.780, *p* < 0.001, *n* = 38) and TMI (*χ*^*2*^ = 13.584, *p* < 0.001, *n* = 38) were significantly higher during normal periods and declined under strong and lingering MHW (Figures 5C and 5D). Conversely, CMI was lowest during normal conditions and significantly higher under MHW exposure (*χ*^*2*^ = 11.124, *p* = 0.004, *n* = 34; Figure 5E).

### Pregnancy shifts energy allocation

Neither phenylalanine δ^15^N values (*χ*^*2*^ = 0.793, *p* = 0.673, *n* = 38) nor Δ^15^N_Glx–Phe_ (*χ*^*2*^ = 1.418, *p* = 0.492, *n* = 38) differed significantly among reproductive stages. The pregnant female (MN-6969) generally exhibited higher GNG and TMI values during confirmed pregnancy than during brief progesterone-elevation periods, and values were similar to or slightly higher (1∼2‰) than those of non-pregnant females, although differences were modest (GNG: *χ*^*2*^ = 15.647, *p* < 0.001, *n* = 38; TMI: *χ*^*2*^ = 5.878, *p* = 0.052, *n* = 38; Figures 5C and 5D). CMI did not differ significantly among reproductive states (*χ*^*2*^ = 1.814, *p* = 0.404, *n* = 34; Figure 5E).

### Interactive effects of stress and reproduction on metabolism

**Figure 6:**
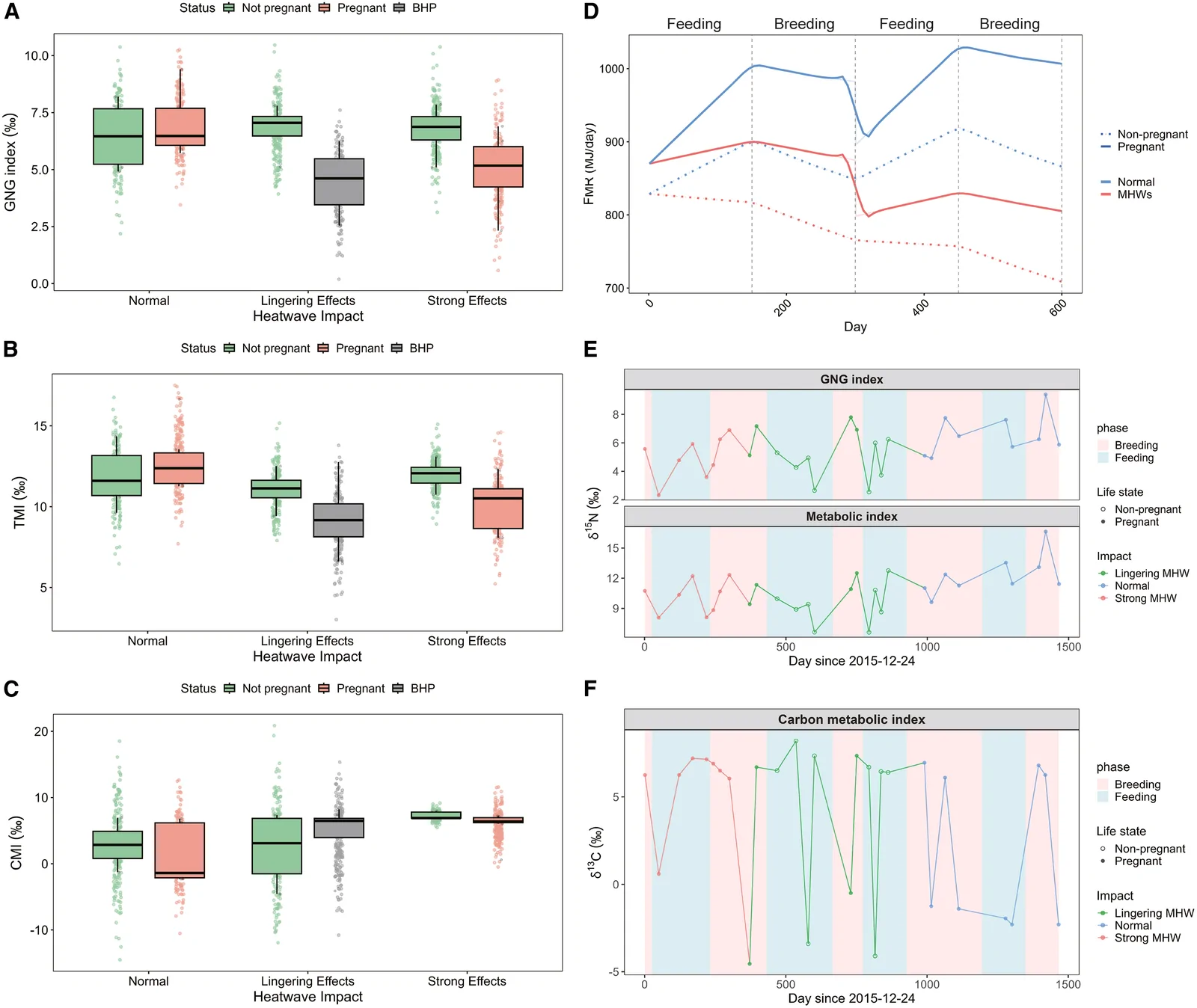
Environmental stress and reproductive status shaped metabolism in humpback whales (A and B) Amino acid-based metabolic indices (GNG index and TMI) across reproductive status (non-pregnant, brief high-progesterone, and pregnant) and heatwave category (normal, lingering, and strong). For both indices, values are generally higher in normal conditions than during MHWs/lingering. (C) Carbon-based metabolic indices (CMI) across reproductive status and heatwave category, indicating differences in lipid utilization among reproductive status and environmental conditions. (D) Predicted field metabolic rate (FMR) through time from the energy-budget model. Pregnancy consistently exhibits higher predicted FMR than non-pregnancy. Within each life state, predicted FMR is higher in normal conditions than during strong MHWs, and the difference (normal − MHW) increases with time. The MHW-driven decline is steeper for pregnancy, indicating a growing energetic penalty under prolonged stress. (E and F) Baleen time series from a single whale, MN-6969, showing metabolic indices across feeding/breeding phases. Periods of heatwave exposure coincide with reduced trophic position and lower metabolic indices, consistent with predicted FMR.

Metabolic indices revealed interactive effects of MHW exposure and reproductive status on nutrient allocation (Figures 6A–6C). Both GNG and TMI values of non-pregnant females were similar across MHW categories (Figures 6B and 6C). The GNG index showed significant effects of reproductive status (*F* = 4.355, *p* = 0.021, *n* = 38) and a significant interaction with MHW category (*F* = 4.277, *p* = 0.047, *n* = 38; Figure 6A). GNG values in non-pregnant females were similar across MHW categories, whereas the pregnant females exhibited a stronger decline during strong and lingering MHWs, widening differences between reproductive states (Figure 6A). A marginally significant interaction was observed between MHW and reproductive status (*F* = 3.984, *p* = 0.054, *n* = 38), while no significant main effects were found for reproductive status (*F* = 1.649, *p* = 0.208, *n* = 38) or MHW categories (*F* = 2.816, *p* = 0.07, *n* = 38) alone (Figure 6B). Pregnant females had lower TMI values during strong and lingering MHW compared with normal periods.

CMI differed significantly among MHW categories (*F* = 4.003, *p* = 0.030, *n* = 34), with the strongest increases during MHWs observed in the pregnant females. Both reproductive groups exhibited lower CMI values under normal conditions than during strong and lingering MHWs (Figure 6C).

Bioenergetic model output further predicted interactive effects of environmental stress and reproductive status on field metabolic rate (FMR) (Figure 6D). Predicted FMR was consistently higher in pregnant than non-pregnant females, and within each reproductive state, predicted FMR was higher under normal conditions than during MHWs. In normal years, both pregnant and non-pregnant females exhibited a seasonal pattern, with elevated predicted FMR on feeding grounds and lower values on breeding grounds. Under strong MHWs, predicted FMR values were initially slightly elevated relative to normal years but declined sharply as the event persisted (∼300 days), ultimately falling below the levels predicted for normal years.

The baleen time series from MN-6969 showed oscillations across feeding and breeding grounds (Figures 6E and 6F). Both GNG and TMI were consistently lower during MHW periods than during normal years, although oscillations between migratory phases were not strongly pronounced. CMI did not exhibit a clear pattern across migratory phases.

**Figure 7:**
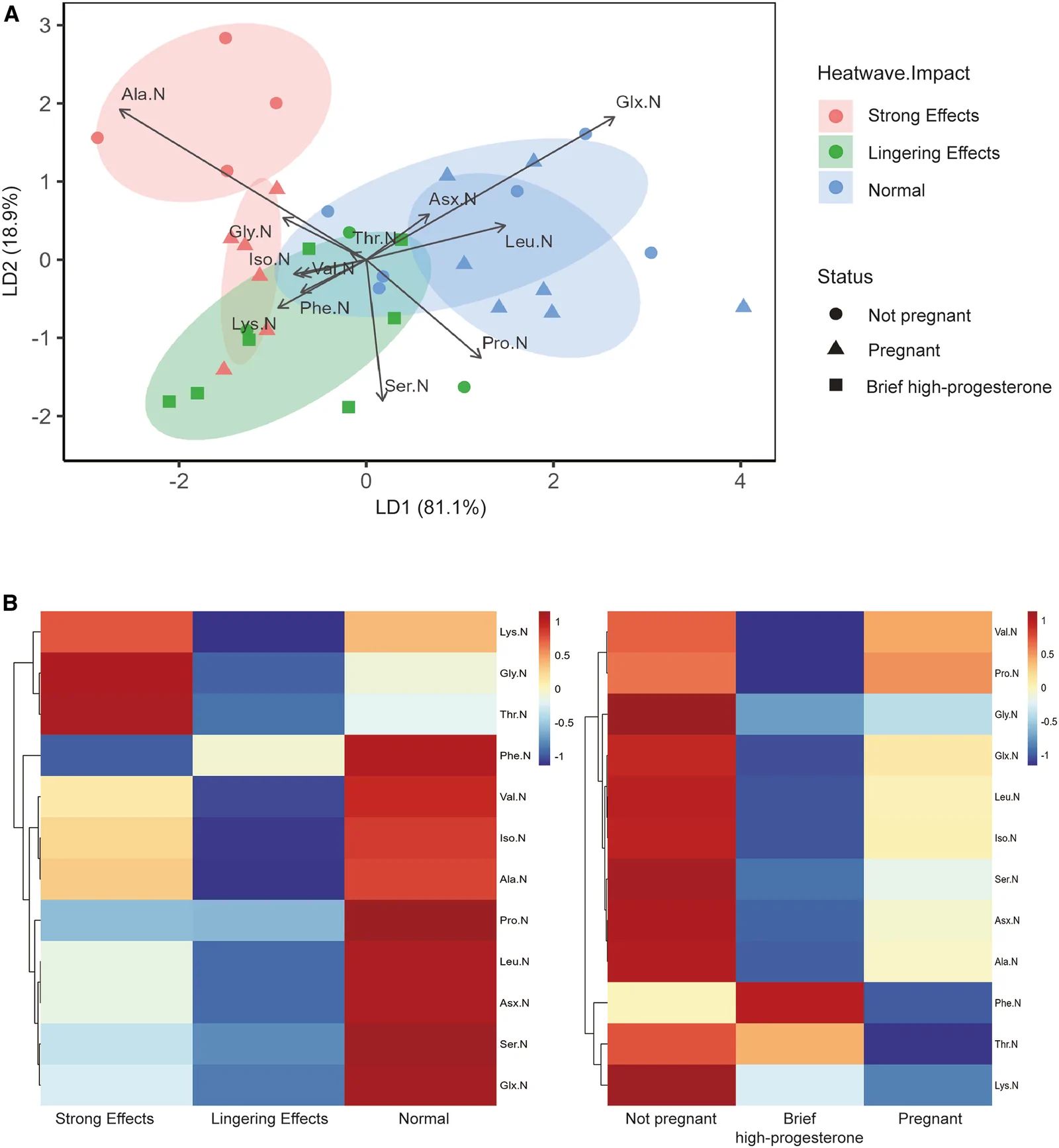
Multivariate separation of amino acid δ^15^N values by environmental stress and reproductive status (A) Linear discriminant analysis (LDA) of amino acid δ^15^N values across heatwave conditions and reproductive states. LD1 (81.1% of variance) separates normal periods (blue) from strong (red) and lingering (green) MHWs, while LD2 (18.9%) separates reproductive status. Vectors indicate amino acids most strongly contributing to group separation. (B) Heatmaps of Z-scored δ^15^N values for individual amino acids across environmental stress categories (left) and reproductive status (right). Red colors indicate higher-than-average values, and blue colors indicate lower-than-average values.

Time series data from all three humpback whales clustered according to MHW exposure and reproductive status (Figure 7). The first linear discriminant (LD1, 81.1%) primarily separated MHW from normal periods, while the second discriminant (LD2, 18.9%) distinguished reproductive states (Figure 7A). Compared with strong and lingering MHW periods, trophic amino acid δ^15^N values were generally higher under normal conditions (Figure 7B). Furthermore, non-pregnant females also exhibited higher δ^15^N values than pregnant females and those with brief progesterone elevations. Together, these results suggest that environmental stress and reproductive status independently structure nutrient utilization patterns in humpback whales.

## Discussion

Our results provide an insight into how humpback whales allocate nutrients and energy under interacting environmental and reproductive pressures. By integrating isotopic biomarkers with bioenergetic modeling, we show that both MHW exposure and reproductive status are associated with shifts in nutrient routing. These findings illustrate how whales may adjust metabolic strategies to balance survival and reproduction during periods of prey limitation imposed by climate-driven events.

Environmental and reproductive stressors exerted stronger influences on nutrient utilization patterns than geolocation, even though bulk δ^15^N values in two individuals exhibited clear seasonal oscillations between breeding and feeding grounds. Baleen δ^15^N values for MN6969 and M-7038 were consistently higher during presumed breeding-ground periods. If these elevated values reflected feeding, δ^15^N values would be expected to decrease, because baseline δ^15^N values in Hawaiian waters are substantially lower than those in the Gulf of Alaska, as demonstrated by the contrast between zooplankton at Station ALOHA and Station Papa. Humpback whales rarely feed in Hawaiian waters.^42^ Instead, the high δ^15^N values in breeding grounds are consistent with expectation for tissue catabolism during fasting.^31^

We initially hypothesized that both bulk and AA isotope values would shift predictably with migratory movements as whales transitioned from exogenous prey to endogenous reserves. However, PCA and LDA analyses did not separate samples by location; instead, they clustered strongly by MHW exposure and reproductive status. This suggests that AA isotope patterns, which primarily reflect internal nutrient routing, were more strongly shaped by physiological state than by geographic position. The MHW period was associated with pronounced metabolic variation, and predicted shifts in nutrient allocation appeared to override seasonal geographic signals.^43^

Across individuals, δ^15^N values of trophic amino acids, along with GNG and TMI, were consistently higher during normal years than during strong or lingering MHWs, whereas CMI values were lower. This pattern is consistent with greater protein turnover and lipid mobilization, supporting elevated metabolic demands. The detailed sighting history of SEAK-5001 provides a rare opportunity to link these biochemical proxies to direct field observations. Approximately 10 days before stranding, SEAK-5001 was observed in a high-energy competitive group off Maui, exhibiting intense surface activity, such as pectoral fin slapping. This behavior, recorded during a non-MHW year, aligns with the elevated GNG index and TMI values in her final baleen segments. These findings contrast with our initial hypothesis that prey-limited MHWs would increase endogenous protein catabolism, expressed as high TMI and GNG and low CMI under strong MHWs. Instead, metabolic indices during normal years reflected efficient nutrient routing in which stored lipid reserves, supplemented by protein turnover, supported elevated metabolic activity. This is consistent with the capacity of large balaenids to accumulate substantial lipid stores—up to ∼45% of body mass in blubber by the end of the feeding season^44^—and subsequently draw on these reserves to fuel fasting, migration, and lactation.^45^^,^^46^^,^^47^

In contrast, prolonged strong and lingering MHWs coincided with reduced predicted FMR, declines in GNG and TMI, and increases in CMI. Together, these shifts indicate suppressed protein turnover and diminished lipid utilization, consistent with metabolic downregulation under stress. This response differs from the classic fasting strategy of pinnipeds, which typically rely on blubber lipids while limiting protein breakdown during predictable fasts such as breeding, molting, or weaning.^48^^,^^49^ Humpback whales experiencing extended MHWs appear unable to sustain this lipid-dominant fasting mode. Prey limitation on feeding grounds likely reduces blubber accumulation, leaving whales with insufficient lipid reserves to mobilize during migration and reproduction. This was apparent in the years following the “Blob” MHW in the Gulf of Alaska, where more than 10% of observed whales on the feeding grounds were noted as being unusually thin between 2016 and 2021.^50^ Consequently, whales exhibited amino acid isotopic patterns consistent with reduced lipid utilization and suppressed protein turnover, indicating a chronically constrained energy state.

Our results differ from patterns observed in elephant seals, where fasting led to substantial ^15^N-enrichment of glucogenic amino acids (e.g., glycine, serine, proline, and aspartic acid) while alanine δ^15^N values declined due to the glucose-alanine cycle.^32^ These patterns reflect a fasting strategy in which lipids remain the primary fuel, but protein is still mobilized to meet glucose and nitrogen demands during prolonged or energetically costly fasting events.^51^ In contrast, humpback whales exposed to MHWs, likely experiencing fasting through reduced prey intake, showed general declines of Δ^15^N_Glx–Phe_ and δ^15^N values across amino acids. This suggests a different metabolic response characterized by reduced protein turnover and lower lipid utilization, likely reflecting inadequate lipid stores during prolonged environmental stress.

The influence of reproductive status on metabolism was weaker than that of environmental stress. We hypothesized that pregnancy would increase reliance on protein catabolism, because fetal growth requires protein as both structural and energetic substrates. Under this scenario, pregnant whales would exhibit higher GNG and TMI values than non-pregnant whales, reflecting greater routing of amino acids into gluconeogenesis.^49^^,^^51^^,^^52^^,^^53^^,^^54^ However, isotopic evidence for increased protein turnover was modest, and trophic amino acid enrichment declined during periods of elevated progesterone. These results suggest that humpback whales may adopt protein-sparing strategies during pregnancy, highlighting the need to consider the combined effects of environmental and physiological stress when interpreting nutrient allocation.

Two intervals of prolonged but brief-duration progesterone elevation were identified between 2017 and 2018, yet their reproductive significance remains uncertain. These peaks did not match the duration or endocrine profiles of full-term pregnancy, and prior analyses showed inconsistent patterns in other reproductive hormones (C.W.C. et al., unpublished data). Distinct shifts in amino acid δ^15^N values during both intervals indicate a physiologically active state distinct from non-pregnant and full-term pregnant periods in the same whale. Together, the hormonal and isotopic evidence suggests that these brief high-progesterone periods represent physiologically distinct reproductive states that may reflect unsuccessful pregnancies or pseudopregnancies, although these possibilities cannot be distinguished with current data. Their timing during the lingering effects of the 2014–2016 MHW, when prey availability and energetic reserves were reduced, is consistent with a potential link between environmental stress and altered reproductive physiology. Pseudopregnancy has been documented in mammals when the corpus luteum remains active following estrous or the loss of an embryo, resulting in continued progesterone production similar in response to an active pregnancy.^55^ Though pseudopregnancy has only been posited in mysticetes, this physiological behavior has been observed in delphinids (C.W.C. et al., unpublished data^56^^,^^57^^,^^58^). This interpretation aligns with long-term monitoring in the Northeast Pacific documenting sharp declines in calf production and reproductive success during and after the same heatwave.^11^^,^^59^ These findings highlight the ability of amino acid isotopes to resolve subtle reproductive transitions.

Nutrient allocation in humpback whales was shaped by interactive effects of reproductive state and environmental stress. Non-pregnant females showed relatively stable GNG and TMI values across MHW categories, suggesting that protein catabolism in this group was less sensitive to environmental variability. However, CMI values increased significantly under strong MHWs, indicating reduced lipid utilization. This pattern is consistent with our bioenergetic model predictions. During prolonged MHWs, declining predicted FMR likely reduces overall energy demands such that protein turnover is maintained at baseline levels while lipid mobilization is suppressed.^48^

Pregnant females exhibited a different response. Both GNG and TMI declined under strong and lingering MHWs, while CMI values increased. This combination indicates suppressed protein turnover and reduced lipid utilization, consistent with metabolic downregulation. Under typical conditions, gestation increases reliance on both lipid reserves and protein catabolism.^49^^,^^52^^,^^53^^,^^54^ Under strong MHWs, however, reduced prey intake and declining predicted FMR may constrain the ability of pregnant females to mobilize stored reserves, resulting in compounded suppression of both protein and lipid metabolism.^60^ These findings highlight how pregnancy magnifies the energetic impact of MHWs by reducing metabolic flexibility at a time of heightened reproductive demand. Interpretation of CMI is limited by δ^13^C data availability for only two individuals, but the observed trends are consistent with shifts toward altered lipid utilization under heatwave stress.

Lingering post-heatwave conditions had stronger impacts on metabolism than strong MHW events. During lingering periods, both metabolic indices and predicted FMR displayed greater variability and steeper declines than during normal conditions. This suggests that recovery from prolonged stress is incomplete, leading to cumulative energetic deficits. Long-term monitoring studies support this interpretation, showing that humpback whale population size and reproductive success required several years to recover following major heatwave events.^60^^,^^61^ Extended disturbances therefore magnify the costs of environmental change, particularly for capital-breeding females whose energy budgets are already constrained by pregnancy.

Together, these findings reveal that humpback whales may adjust nutrient allocation strategies in response to environmental stress and reproductive demand, but that prolonged heatwaves intensify energetic penalties and limit recovery capacity. As MHWs become more frequent and persistent under climate change, the energetic buffer that enables long-distance migration and reproduction may be increasingly compromised. These results provide insight into potential links between climate extremes and nutrient utilization strategies in capital breeders, with implications for population resilience and ecosystem dynamics.

### Limitations of the study

Several limitations should be acknowledged despite the high-resolution, long-term nature of these data. First, the sample size is restricted (*n* = 3), and the reproductive stages did not consistently overlap across MHW intensities. This limits our ability to fully disentangle whether observed metabolic shifts were driven primarily by MHW exposure or by intrinsic physiological differences among individuals. North Pacific humpback whales comprise four distinct population segments (DPSs), including the endangered Western North Pacific DPS and Central America DPS, that migrate annually between low-latitude breeding grounds and shared high-latitude foraging areas. During and after the 2014–2016 MHW, the Hawaii DPS exhibited pronounced physiological disruption and reduced reproductive output, whereas whales from the Mexico and Central America DPS showed comparatively stable demographic trajectories despite experiencing the same basin-scale anomaly.^61^^,^^62^ Expanding amino acid isotope and hormonal analyses to whales from additional Pacific DPS would help to identify critical gaps in understanding how environmental variability, prey limitation, and anthropogenic pressures translate into physiological stress and demographic outcomes across humpback whale populations.

Second, the bioenergetic model relies on assumption-based parameters (e.g., specific caloric intake and migration costs) derived from existing literature. Although model outputs are validated against empirical metabolic ranges for large cetaceans, the absence of direct foraging data during the MHW events means these results should be interpreted as mechanistic simulations rather than direct measurements. Nonetheless, given the difficulty of obtaining continuous, multi-year biochemical records from large whales, these data provide rare insight into the physiological plasticity of baleen whales facing climatic anomalies.

## Resource availability

### Lead contact

Further information and requests for resources and reagents should be directed to and will be fulfilled by the lead contact, Brian N. Popp (popp@hawaii.edu).

### Materials availability

This study did not generate new unique reagents.

### Data and code availability

• Bulk tissue and amino acid isotope datasets along with progesterone are available in Zenodo (https://doi.org/10.5281/zenodo.18251474).
• The bioenergetic model code used in this study is archived in Zenodo (https://doi.org/10.5281/zenodo.18251433).
• DNA profiles and microsatellite data for individual SEAK-5001 are available in GenBank under accession code KF477244–KF477271.

## Statement of inclusion

Our study brings together authors from different countries. We worked closely with local stranding networks and management agencies in Hawaii, ensuring that the biological samples used were collected under strict federal permits and with the support of local staff and volunteers. Our study includes researchers at different career stages, from PhD candidates to senior faculty, and we have prioritized equitable contribution and credit throughout the conceptualization, data collection, and manuscript preparation phases.

## Acknowledgments

We thank the Office of Naval Research Marine Mammals and Biology Program award N00014-21-1-2642. Stranding response and baleen collection were supported by the NOAA National Marine Fisheries Service (NMFS) John H. Prescott Marine Mammal Rescue Assistance Grant program over many years and was conducted under NOAA/NMFS permit 18786. We thank support from the National Science and Technology Council, Taiwan (NSTC 114-2611-M-056-001) for covering the article processing charge. We would also like to thank the staff and volunteers of the Health and Stranding lab for their assistance with stranding response and animal necropsies. This is the 10.13039/100008783University of Hawai'i at Mānoa, SOEST contribution number 12159.

## Author contributions

C.-T.C. and B.N.P. designed and developed the concept of this study. K.L.W. and B.N.P. secured funding for this project. C.W.C. and K.L.W. conducted sample collections and contributed to discussions that shaped this study. D.S. and S.B. conducted the DNA experiment. C.-T.C. and N.J.W. conducted the isotope experiment. C.W.C. conducted the hormone extraction and analyses. The manuscript was drafted by C.-T.C. All co-authors contributed critically to the drafts and gave final approval for publication.

## Declaration of interests

The authors declare no conflict of interest.

## Declaration of generative AI and AI-assisted technologies in the writing process

During the preparation of this work, the authors used ChatGPT (OpenAI) to assist with language editing, grammar checking, and code troubleshooting. The authors reviewed and edited the generated content as needed and take full responsibility for the content of the publication.

## STAR★Methods

### Key resources table

**Table:** 

REAGENT or RESOURCE	SOURCE	IDENTIFIER
**Biological samples**		

Humpback whale baleen plates	This paper	MN-7038, MN-6969, SEAK-5001

**Critical commercial assays**		

Hormone enzyme immunoassay kit	Arbor Assays	Cat# K025-H5; RRID: AB_2924718

**Deposited data**		

Baleen isotope and progesterone dataset	This paper; Clifton et al., unpublished data	https://doi.org/10.5281/zenodo.18251474
Bioenergetic model code	This paper	https://doi.org/10.5281/zenodo.18251433
DNA profiles and microsatellite data	Baker et al. (2013)	GenBank accession code: KF477244-KF477271

**Software and algorithms**		

R	R Core Team	Version 4.0.4
lme4	Bates et al. 2015	https://cran.r-project.org/package=lme4
effectsize	Ben-Shachar et al. 2020	https://cran.r-project.org/web/packages/effectsize
nlme	Pinheiro et al. 2023	https://cran.r-project.org/web/packages/nlme
NOAA Coral Reef Watch marine heatwave data	NOAA Coral Reef Watch	https://coralreefwatch.noaa.gov/product/marine_heatwave/
GENEMAPPER	Thermo Fisher Scientific	Version 3.7

**Other**		

Delta V Plus isotope ratio mass spectrometer	Thermo Fisher Scientific	NA
Trace GC gas chromatograph	Thermo Fisher Scientific	NA
Costech ECS 4010 Elemental Combustion System	Costech Analytical Technologies	NA

### Experimental model and study participant details

#### Stranded humpback whales

This study did not use live experimental animals, cell lines, or genetically defined model organisms. Baleen plates were obtained from three deceased stranded female humpback whales (*Megaptera novaeangliae*; MN-7038, MN-6969, and SEAK-5001) and analyzed retrospectively. No live animals were handled for this study. Stranded baleen collection were conducted under NOAA/NMFS permit 18786.

### Method details

#### Ethics statement and permits

All research activities, including sample collection and processing, were conducted under appropriate institutional ethics guidelines and government permits. Stranding response and baleen collection was collected under NOAA/NMFS permit 18786.

#### Sample collection, DNA profiling, and baleen preparation

Baleen plates were collected from three stranded female humpback whales (IDs: MN-7038, MN-6969, SEAK-5001) and stored at −20°C until analysis. All animals were deceased at time of stranding (Table 1) and were recovered from different locations across Hawaii, as depicted in the sub-panel of Figure 1. MN-7038 stranded at ʻĀhihi, Maui, on 29 December 2016 (baleen length ∼65 cm; body length unknown). No detailed morphometric data is available for this individual. MN-6969 was recovered from Hale O Lono, Molokaʻi, on 21 January 2020 (baleen length ∼75 cm; body length 13.4 m) and was pregnant with a full-term female fetus at the time of stranding. SEAK-5001 stranded at Waimanalo, Oʻahu, on 12 April 2021 (baleen length ∼65 cm; approximate body length 10.6 m). The approximate length was recorded while the animal was still shifting due to wave action.

DNA extracted from gum tissue at the proximal end (newest growth) of the baleen plate for each individual. A comprehensive DNA profile – including sex, mitochondrial DNA control region haplotype, and 10 nuclear microsatellite loci (Table S3) – was generated following established protocols for North Pacific humpback whales^37^ and compared to a DNA register of over 4,200 humpback whales sampled across the North Pacific.

Baleen from MN-6969 had been previously analyzed for concentrations of androstenedione, 17β-estradiol, progesterone, and testosterone (Clifton et al., unpublished data). The same preparatory and analytical procedures were applied to baleen collected from SEAK-5001 and MN-7038. To prepare the plates for analysis, excess gingival tissue was removed, and plates were washed with soap, clamped and dried for one week. Baleen was sampled at 1-cm intervals along its growth axis, beginning at the gingival margin (defined as position 0). At each increment, a rotary tool (Dremel 200) was used to drill transversely through the plate. Tools and baleen plates were rinsed three times with ethanol between increments to prevent cross-contamination. Approximately 300 mg of powder was collected per increment, transferred to tubes containing ceramic beads, and homogenized using a bead mill (Bead Ruptor 12, Omni International) to produce a uniform sample for isotopic and hormone analyses.

#### Stable isotope analysis of bulk tissue

For bulk isotope analysis, 0.4–0.8 mg of baleen powder from SEAK-5001 and MN-7038 was weighed into ultra-clean tin capsules. The values of δ^13^C and δ^15^N were measured using an elemental analyzer (Costech ECS 4010 Elemental Combustion System) coupled to an isotope ratio mass spectrometer (Thermo-Delta V Advantage). The isotope values are reported in standard per mil (‰) notation, relative to Vienna Pee Dee Belemnite (VPDB) for carbon and atmospheric N_2_ for nitrogen. Analytical error, based on repeated measurements of reference materials was <0.2‰ for both δ^13^C and δ^15^N.

#### Progesterone extraction and analysis

Baleen progesterone extraction from SEAK-5001 and MN-7038 followed established protocols (Clifton et al., unpublished data,^20^^,^^21^^,^^34^). Briefly, ∼100 mg of powdered baleen was combined with 6 mL methanol in 12-mL glass tubes and mixed for 4 h at 700 rpm on a Glas-Col digital pulse mixer. Samples were centrifuged for 20 min, and the supernatant was transferred volumetrically into 20-mL scintillation vials. Extracts were dried overnight in a vacuum concentrator (SpeedVac SPD210, Thermo Fisher Scientific). Dried extracts were reconstituted in 0.5 mL assay buffer (Arbor Assays, Ann Arbor, MI) and stored at −80°C prior to analysis.

#### Nitrogen and carbon isotope analysis of AAs

Sample preparation for CSIA-AA followed the methods from previous studies.^63^^,^^64^ Approximately 10–15 mg of homogenized baleen powder was hydrolyzed, esterified, and trifluoroacetylated. The δ^15^N values of amino acids were measured using a Delta V Plus mass spectrometer interfaced to a Trace GC gas chromatograph. The δ^13^C values of amino acids were determined using a Thermo Scientific MAT 253 mass spectrometer coupled to a Thermo Scientific Trace GC Ultra gas chromatograph. The measured δ^15^N values were corrected via linear regression based on the relationship between measured and known isotopic values of 14 amino acid reference compounds. The measured δ^13^C values were corrected relative to this AA suite following previous study.^64^ All samples were analyzed in triplicate. Standard deviation for triplicate injections averaged 0.40‰ (±0.40‰) on δ^15^N values and 0.47‰ (±0.40‰) on δ^13^C values. Notably, carbon isotope analysis of amino acids was conducted on only two humpback whales, MN-7038 and MN-6969.

#### Classification of life history and environmental stress

##### Chronological reconstruction of baleen records

Baleen growth rates were inferred by interpreting isotopic oscillations and aligning them with temporal markers such as time and date of stranding or expected foraging–fasting cycles.^33^^,^^36^ Growth rate estimates were derived from sequential maxima and minima in δ^15^N values along the baleen profile, with approximate dates assigned relative to stranding event.

##### Determination of MHWs

MHW conditions were classified into three categories: (i) strong MHW (2015 to early 2016), characterized by the persistent anomaly of the prominent Northeast Pacific MHW (known as ‘The Blob’), where sea surface temperature (SST) anomalies along the migration pathways between the Gulf of Alaska and the Hawaiian Islands continuously exceeded Category 3 (Severe) or Category 4 (Extreme) thresholds^4^ (https://coralreefwatch.noaa.gov/product/marine_heatwave/); (ii) lingering effect (late 2016 to mid 2018), defined as the period immediately following the dissipation of ‘The Blob’ in late 2016. Although physical SST anomalies subsided, delayed ecological repercussions persisted, characterized by a severely depressed krill biomass in the Gulf of Alaska and documented collapses in baleen whale reproductive success, such as a drastic reduction in whale calf production^11^^,^^15^^,^^65^^,^^66^; and (iii) normal condition (late 2018, 2019, 2021). While a minor SST warming event occurred around 2019–2020, the krill biomass did not significantly decrease in the marine ecosystems, so we classified 2019–2020 as normal. Finally, we applied linear discriminant analysis (LDA) to amino acid δ^15^N values – after controlling for reproductive status – to evaluate whether isotopic composition supported or refined these MHW classifications (Figure S3).

##### Determination of reproductive status

Estimated dates derived from bulk stable isotopes, progesterone concentrations, and life history information were used to identify reproductive status within each baleen record. Detailed hormone and bulk isotope data interpretation for MN-6969 can be found in a previous study (Clifton et al., unpublished data). Sample status was categorized into three categories: (i) pregnant: samples occurring during a known pregnancy with a confirmed fetus, or during a presumed pregnancy based on progesterone concentrations continuously remaining above baseline for >11 months; (ii) non-pregnant: samples with progesterone concentrations near baseline; and (iii) brief-duration high-progesterone: samples with progesterone level consistently above baseline but for a duration insufficient to represent a successful pregnancy (<11 months).

Following reproductive status assignment, a subset of samples was selected for CSIA-AA to represent the identified categories. We used LDA of amino acid δ^15^N values, after controlling for MHW effects, to evaluate whether isotopic composition independently classified reproductive status. We first tested isotopic differences between the pregnant and non-pregnant samples to establish a baseline for reproductive distinction. Once this separation was confirmed, the brief-duration high-progesterone samples were added as a test group. Samples that did not cluster with either pregnant or non-pregnant categories were interpreted as representing a distinct physiological state.

##### Determination of migratory phases and locations

The migratory phases were assigned to each baleen segment based on chronological reconstruction described above. Following the established migratory patterns of North Pacific humpback whales,^67^ we allocated segments to two primary regions based on the estimated calendar month: (1) Feeding grounds (Gulf of Alaska), spanning from approximately June to October (summer/autumn), and (2) Breeding grounds (Hawaiian Islands), spanning from January to March (winter). Assignments were based on estimated calendar dates derived from baleen growth chronology Segments corresponding to the transition periods (April–May and November–December) were categorized as migratory phases. Differences in bulk tissue δ^15^N values between feeding ground and breeding ground were evaluated using a non-parametric Wilcoxon rank-sum test.

#### Data analysis

##### Estimation of partial η^2^ effect sizes of each factor

To quantify the relative influence of environmental stress, reproductive status, and location on amino acid δ^15^N variation, we calculated partial η^2^ (eta-squared) effect sizes from linear mixed-effects model (LMM). For each amino acid, we fitted an LMM with δ^15^N as the response variable and marine heatwave category (normal, strong, lingering), reproductive status (non-pregnant, brief duration high-progesterone, pregnant), and location (feeding vs. breeding grounds) as fixed effects, with individual whale ID as a random effect to account for repeated measures within individuals. The models were fitted using the lmer() function in the lme4 package^68^ in R 4.0.4. Partial η^2^ values were computed using the effectsize package^69^ in the following equation:

(Equation 1) $${1.\;\eta }_{p}^{2}=\frac{{SS}_{effect}}{{SS}_{effect}+{SS}_{error}}$$

where SS_effect_ is the sum of squares attributable to a given factor, and SS_error_ is the residual sum of squares. Partial η^2^ values were interpreted following conventional thresholds^70^: small (≥0.01), medium (≥0.06), and large (≥0.14).

##### Isotopic indices and linear mixed-effects modeling

We examined nutrient allocation under varying environmental stress (normal, lingering, strong MHWs), reproductive status (pregnant, brief high-progesterone, non-pregnant), and migratory phase (feeding ground vs. breeding ground) using isotopic indices from amino acid δ^15^N and δ^13^C values.

Phenylalanine (Phe) and lysine (Lys) were used as source amino acids, representing the δ^15^N baseline values of the local food web. The difference in the δ^15^N values of glutamic acid and phenylalanine (Δ^15^N_Glx–Phe_) served as an indicator of effective food-chain length and trophic position. We defined three additional indices to quantify metabolic routing and nutrient utilization:

(1) Gluconeogenic index (GNG index):

(Equation 2) $$GNG={\bar{\delta^{15}N}}_{\left(Gly,Ser,Pro,Asx\right)}{-\bar{\delta^{15}N}}_{\left(Phe,Lys\right)}$$

This index reflects the extent to which whales route amino acids into gluconeogenesis and was calculated as the isotopic difference between a group of AAs that participant in gluconeogenesis (Gly, Ser, Pro, and Asp) and those that typically undergo minimal isotopic fractionation (Phe, Lys).^71^ While Pro and Asp are identified as classic trophic AAs that undergo extensive transamination, their carbon are heavily mobilized as primary substrates in the gluconeogenic pathway under high-protein or fasting states.^72^ Thus, this index reflects the physiological extent to which whales convert either dietary (exogenous) or body (endogenous) proteins into glucose to meet their high metabolic demands.^32^ While standard fasting (Stage II) is characterized by protein-sparing and a reliance on lipid oxidation, an elevated GNG index under extreme conditions reflects a metabolic shift toward increased reliance on protein-derived carbon backbones to maintain blood glucose. Therefore, higher indices are interpreted as an increased contribution of amino acids to the gluconeogenic pool, which—under prolonged negative energy balance caused by reduced energy acquisition or when lipid stores are depleted—may indicate a transition toward Stage III fasting and overall increased protein catabolism.

(2) Trophic metabolic index (TMI):

(Equation 3) $$\text{TMI}={\bar{\delta^{15}N}}_{\left(Glx,Ala,Asx,Pro,Leu,Val,Iso\right)}{-\bar{\delta^{15}N}}_{\left(Phe,Lys\right)}$$

The TMI reflects the relative intensity of nitrogen cycling and protein turnover, as the differences of trophic amino acids with strong participation in the metabolic nitrogen pool and source amino acids with minimal fractionation among the metabolic process.^31^ Because trophic amino acids become enriched in ^15^N during transamination and nitrogen recycling, elevated TMI values indicate increased nitrogen flux through protein metabolism, consistent with enhanced amino acid turnover and potentially greater reliance on endogenous protein catabolism.

(3) Carbon metabolic index (CMI)

(Equation 4) $$\text{CMI}={\bar{\delta^{15}C}}_{\left(Glx,Asx\right)}{-\bar{\delta^{15}C}}_{\left(Phe,Lys\right)}$$

The CMI represents relative routing of carbon between non-essential and essential amino acids. Lower CMI values are consistent with greater incorporation of ^13^C-depleted carbon into non-essential amino acids, suggesting increased routing of lipid-derived carbon during metabolism.^17^ Because lipids are generally more ^13^C-depleted than protein and carbohydrate sources, lower CMI values likely reflect greater reliance on lipid reserves to sustain normal energetic demands. Conversely, elevated CMI values may indicate reduced lipid mobilization associated with metabolic downregulation under prolonged environmental stress.

For calculating CMI, only two stranded humpback whales (MN-7038 and MN-6969) were included. The non-pregnant individual from a normal year (SEAK-5001) was excluded because δ^13^C values were not measured for this specimen.

We analyzed the effects of environmental stress and reproductive status on δ^15^N values and derived indices using linear mixed-effects models (LMMs) implemented in nlme.^73^ The general model structure was:

(Equation 5) $$Y_{i}=\beta_{0}+\beta_{1}\left(MHW\right)+\beta_{2}\left(Reproductivestatus\right)+\left(1|\text{Individual}\right)+\varepsilon$$

where Y_i_ represents δ^15^N_Phe_, Δ^15^N_Glx–Phe_, or a derived isotopic index; fixed effects included MHW intensity and reproductive status; and whale ID was treated as a random intercept. To account for potential pseudoreplication and temporal autocorrelation among sequential baleen segments within each individual, a first-order autoregressive (AR1) correlation structure was incorporated into the LMM. The significance of fixed effects was assessed using Type II Wald χ^2^ tests (car package). Following the LMM, post-hoc pairwise comparisons were conducted using estimated marginal means (emmeans package) with Tukey’s HSD test to identify specific differences between categorical main effects.

#### Field metabolic rate modeling

Field metabolic rates (FMR) of humpback whales were predicted using a bioenergetic model adapted from Braithwaite et al. 16 and McHuron et al. 53. The model incorporated environmental conditions (strong MHWs vs. normal), reproductive status (pregnant vs. non-pregnant), and migratory regions (feeding vs. breeding grounds). Daily energy balance (E_Balance_) was estimated over sequential 300 days annual cycles as follow:

$$E_{Balance}=I\times \text{AE}-\left(E_{BMR}+E_{\mathrm{COT}}+E_{Preg}\right)$$

where I is daily gross energy intake, AE is assimilation efficiency^74^ (set at 0.9), E_BMR_ is basal metabolic rate, E_COT_ is the cost of transport, and E_Preg_ is the cost of pregnancy. The basal metabolic rate (E_BMR_) followed Kleiber’s Law (0.293 M^0.75^ MJ/Day), consistent with a previous humpback whale energetic study.^16^ The cost of transport (E_COT_) was calculated based on body mass (M) and swimming speed (U), where E_COT_ = (4.5 ·10^−4^ · M^0.85^ · U^2^)/1000.^16^ Following Braithwaite et al. 16, an optimal migration speed of 1.1 m/s was assumed to minimize total energy expenditure. To account for the growth of the fetus in capital breeders, we incorporated a time-varying pregnancy cost. This cost was modeled as a dynamic multiplier that increases linearly from 5% to 15% of the baseline E_BMR_ throughout the gestational period.^54^ We simulated the impact of MHWs by introducing a suppression factor (β_feedfrac_ = −0.75) to the feeding success on high-latitude foraging grounds. This 75% reduction in energy intake that was consistent with the dramatic declines in prey availability (e.g., krill and herring) observed during severe thermal anomalies.^59^ The model was run in R 4.0.4 using a daily time-step. Energy reserves were stored as blubber with a lipid density of 39.3 MJ/kg.^75^

### Quantification and statistical analysis

#### Analytical quantification

Raw δ^13^C and δ^15^N bulk baleen values were calibrated against international standards (VPDB for carbon and atmospheric N_2_ for nitrogen). Analytical precision was maintained at < 0.2‰ through repeated measurements of internal reference materials every 10 samples. For CSIA-AA, the measured δ^15^N values were corrected via linear regression based on the relationship between measured and known isotopic values of 14 amino acid reference compounds. The measured δ^13^C values were corrected relative to this AA suite following previous work.^64^ All CSIA-AA samples were analyzed in triplicate. Standard deviation for triplicate injections averaged 0.40‰ (±0.40‰ S.D.) on δ^15^N values and 0.47‰ (±0.40‰ S.D.) on δ^13^C values. Hormone extracts were also analyzed in triplicate, with a sample coefficient of variation (CV) threshold of 10% for quality control. The average intra-assay variation was 5.0%, while average inter-assay variation was 9.0% (Clifton et al., unpublished data).

Microsatellite alleles were sized and binned using GENEMAPPER v3.7. This software also assigns a quality scores based on peak height and shape. All automated bins, regardless of quality score, were manually checked to ensure genotyping accuracy.

#### Statistical analyses

All statistical analyses were conducted in R version 4.0.4. Significance was set at *p* < 0.05. Microsatellite alleles were sized and binned using GENEMAPPER v3.7.

### Additional resources

This study did not generate or contribute to a new website, forum, or clinical trial resource.
