# Supplementary material for: Marine heatwaves suppress nutrient utilization and reproductive flexibility in humpback whales
Source: iScience. 2026 Aug 19;29(9):117181. doi: 10.1016/j.isci.2026.117181 (PMC13521090; doi:10.1016/j.isci.2026.117181)
Supplement: Supplementary file 1 [file mmc1.pdf]

iScience, Volume *29*

Supplemental information

Marine heatwaves suppress nutrient utilization and reproductive flexibility in humpback whales

Ching-Tsun Chang, Cody W. Clifton, Kristi L. West, Debbie Steel, Scott Baker, Natalie J. Wallsgrove, and Brian N. Popp

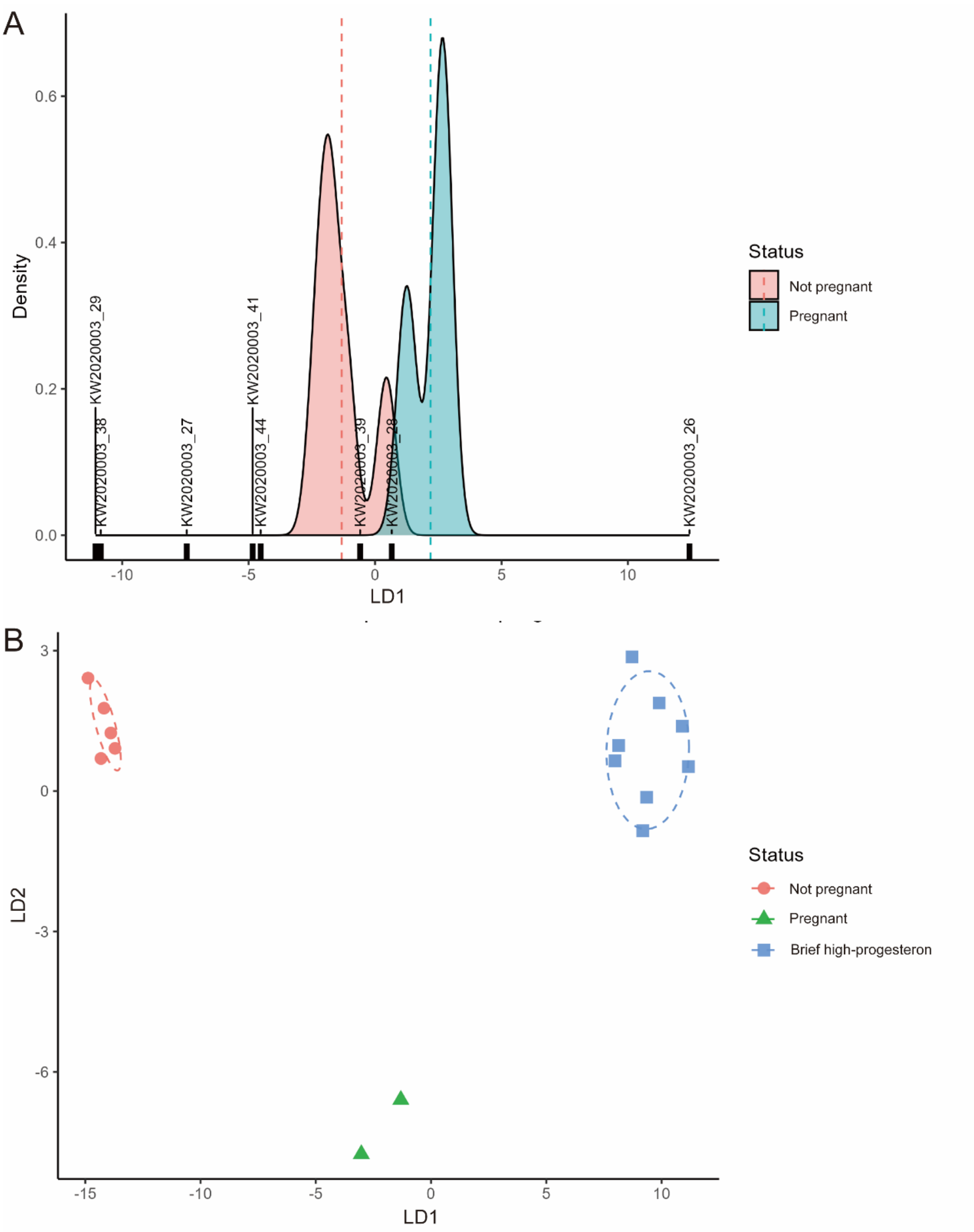

Figure S1. Classification of reproduction status using amino-acids $\delta^{15}N$ values after controlling for marine heatwaves effects. (A) Density distributions of linear discriminant (LD1) scores for samples with known reproductive status (red = Not pregnant; blue = Pregnant). Dashed vertical lines indicate the group means. Black tick marks denote samples from prolonged high-progesterone intervals, most of which fall outside the LD1 ranges of the two known groups. (B) Linear discriminant analysis (LDA) showing separation among non-pregnant (red circles), pregnant (green triangles), and brief high-progesterone (blue squares) samples after eliminating the marine heatwaves effects.

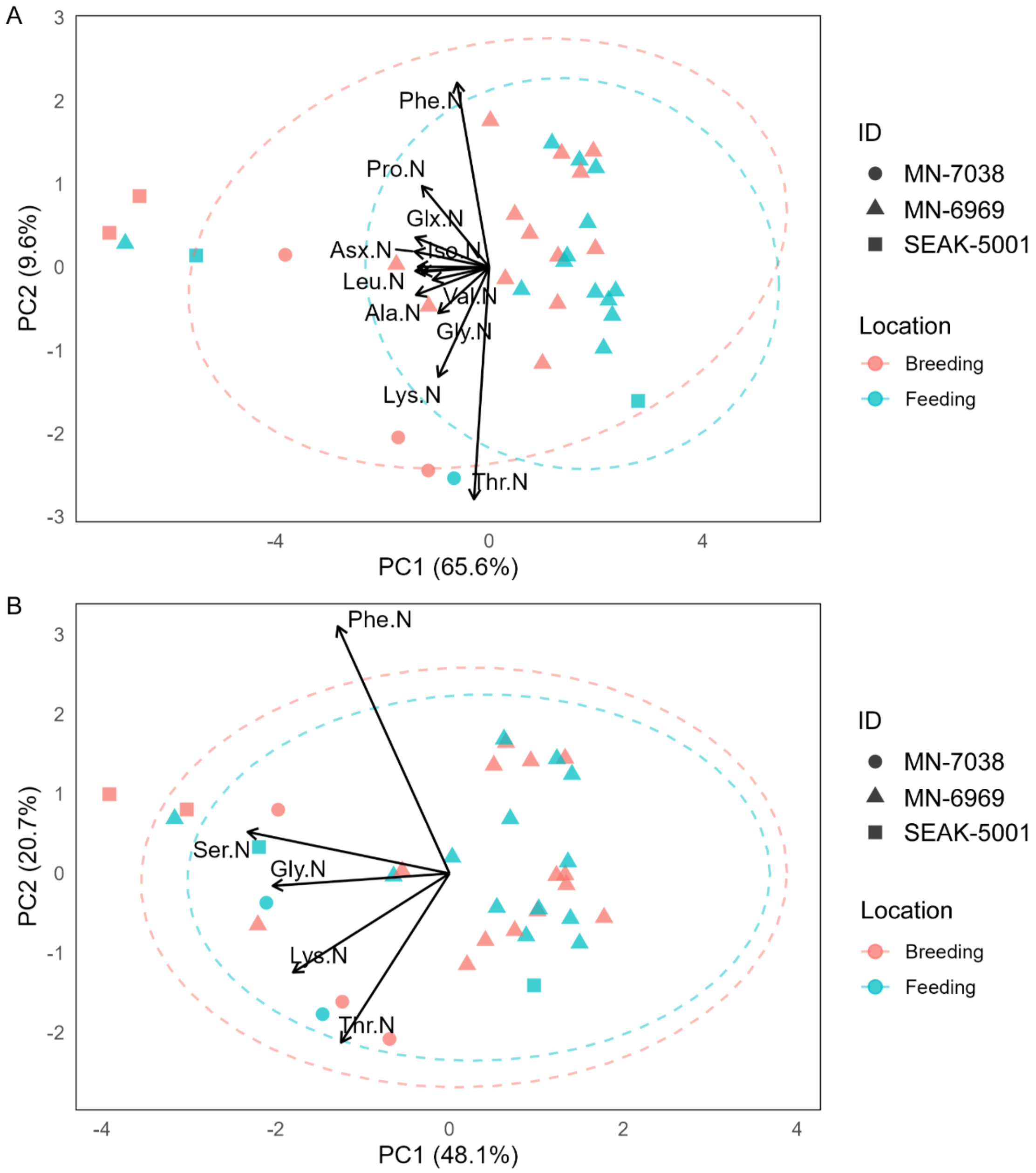

Figure S2. Principal component analysis (PCA) of amino-acid δ^{15}N values between feeding and breeding grounds. (A) PCA including all amino acids. (B) PCA including only source amino acids. Substantial overlap between groups indicates limited separation by migratory phase.

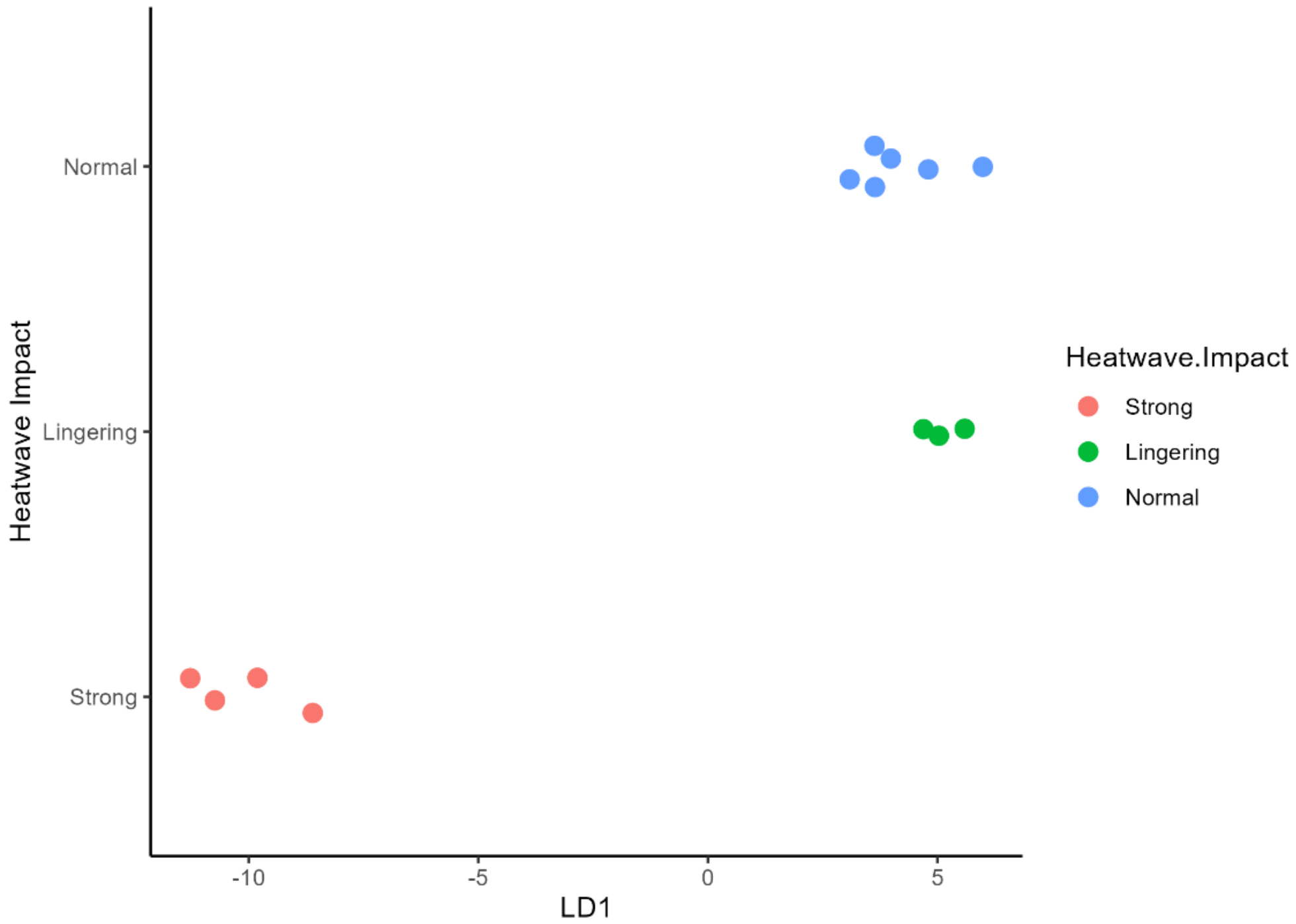

Figure S3 Classification of marine heatwave (MHW) status using amino-acids $\delta^{15}N$ values after controlling for reproductive effects. Linear discriminant analysis (LDA) showing separation among samples assigned to strong MHW effects (red), lingering MHW effects (blue), and normal conditions (green) samples after eliminating the reproductive effects. To isolate environmental influences, only samples from non-pregnant stages were included.

Table S1 Statistical results of δ^{15}N variations across marine heatwave categories. Summary of *F* statistics, degrees of freedom, and *p* values from ANOVA tests evaluating the effects of marine heatwave (MHW) intensity on δ^{15}N values of bulk tissue and individual amino acids. Significant differences are indicated by $p < 0.05$ (*) and $p < 0.01$ (**).

δ^{15}N	*F* values	Degrees of freedom	*P* values
Bulk tissue	6.092	35	0.005**
Alanine	4.272	33	0.022*
Aspartic acid	5.417	35	0.009**
Proline	7.847	35	0.002**
Glutamic acid	8.709	35	0.000**
Glycine	0.272	35	0.764
Isoleucine	3.125	34	0.057
Leucine	5.893	35	0.252
Lysine	0.081	34	0.923
Phenylalanine	0.928	35	0.405
Serine	4.032	35	0.027*
Threonine	0.508	34	0.606
Valine	3.827	33	0.032*

Table S2 Statistical results of δ^{13}C variations across marine heatwave categories. Summary of *F* statistics, degrees of freedom, and *p* values from ANOVA tests evaluating the effects of Marine heatwave (MHW) intensity on δ^{13}C values of bulk tissue and individual amino acids. Significant differences are indicated by $p < 0.05$ (*) and $p < 0.01$ (**).

δ^{13}C	*F* values	Degrees of freedom	*P* values
Bulk tissue	1.710	32	0.097
Alanine	0.189	31	0.828
Aspartic acid	1.046	30	0.364
Proline	3.873	31	0.032*
Glutamic acid	3.700	31	0.036*
Glycine	0.928	31	0.406
Isoleucine	0.604	29	0.553
Leucine	0.202	30	0.818
Lysine	1.064	30	0.358
Phenylalanine	3.071	31	0.061
Serine	0.524	30	0.598
Threonine	0.327	31	0.723
Valine	0.084	31	0.920

Table S3 DNA profiles for MN-6969 and SEAK-5001. MN-6969 was recovered from Hale O Lono, Molokaʻi, on 21 January 2020. SEAK-5001 stranded at Waimanalo, Oʻahu, on 12 April 2021. No data is available for MN-7038.

	**Genetic ID**	gHI_KW2020003	gNBC05-5310
	**Whale ID**	MN-6969	SEAK-5001
	**Year**	2020	2021
	**Region**	Hawaii	Hawaii
	**Sex**	F	F
	**mtDNA Haplotype**	F2	A-
	**GenBank Reference**	KF477266	KF477245
**Microsatellite loci**	Ev14	131	131
	Ev14	137	131
	Ev37	192	192
	Ev37	200	196
	Ev96	161	157
	Ev96	165	161
	GATA28	147	147
	GATA28	187	147
	GATA417	214	195
	GATA417	222	206
	GT211	110	108
	GT211	116	110
	GT23	111	111
	GT23	111	115
	GT575	149	157
	GT575	153	163
	rw4-10	196	196
	rw4-10	206	196
	rw48	112	114
	rw48	116	120
	Ev1	123	123
	Ev1	123	127
	Ev104	0	151
	Ev104	0	151
	Ev21	111	111
	Ev21	117	115
	Ev94	206	206
	Ev94	212	212
	rw31	116	106
	rw31	118	114
	Ev14	131	131
